# Resveratrol has an Overall Neuroprotective Role in Ischemic Stroke: A Meta-Analysis in Rodents

**DOI:** 10.3389/fphar.2021.795409

**Published:** 2021-12-20

**Authors:** Jianyang Liu, Jialin He, Yan Huang, Zhiping Hu

**Affiliations:** ^1^ Department of Neurology, The Second Xiangya Hospital, Central South University, Changsha, China; ^2^ National Health Commission Key Laboratory of Birth Defects Research, Prevention, and Treatment, Hunan Provincial Maternal and Child Health Care Hospital, Changsha, China

**Keywords:** resveratrol, ischemic stroke, meta-analysis, neuroprotection, therapy

## Abstract

**Background:** Resveratrol, a natural polyphenolic phytoalexin, is broadly presented in dietary sources. Previous research has suggested its potential neuroprotective effects on ischemic stroke animal models. However, these results have been disputable. Here, we conducted a meta-analysis to comprehensively evaluate the effect of resveratrol treatment in ischemic stroke rodent models.

**Objective:** To comprehensively evaluate the effect of resveratrol treatment in ischemic stroke rodent models.

**Methods:** A literature search of the databases Pubmed, Embase, and Web of science identified 564 studies that were subjected to pre-defined inclusion criteria. 54 studies were included and analyzed using a random-effects model to calculate the standardized mean difference (SMD) with corresponding confidence interval (CI).

**Results:** As compared with controls, resveratrol significantly decreased infarct volume (SMD −4.34; 95% CI −4.98 to −3.69; *p* < 0.001) and the neurobehavioral score (SMD −2.26; 95% CI −2.86 to −1.67; *p* < 0.001) in rodents with ischemic stroke. Quality assessment was performed using a 10-item checklist. Studies quality scores ranged from 3 to 8, with a mean value of 5.94. In the stratified analysis, a significant decrease of infarct volume and the neurobehavioral score was achieved in resveratrol sub-groups with a dosage of 20–50 mg/kg. In the meta-regression analysis, the impact of the delivery route on an outcome is the possible source of high heterogeneity.

**Conclusion:** Generally, resveratrol treatment presented neuroprotective effects in ischemic stroke models. Furthermore, this study can direct future preclinical and clinical trials, with important implications for human health.

## Introduction

Ischemic stroke is one of the major causes of morbidity and long-term disability in the worldwide population. At present, intravenous thrombolysis and endovascular thrombectomy are effective therapy within a limited time window (Fisher and Saver, 2015). Owing to the poor regenerative ability of the adult brain, stroke-induced neuronal injury is permanent and results in a long-term neurological deficiency. Therefore, various effective therapy to reduce post-ischemic neuronal cell or tissue loss remain in further research.

Resveratrol (3,5,4′-trihydroxystilbene) (PubChem CID: 445154) is a natural estrogen-like phytosterol that mainly is found in grapes, blueberries, peanuts, red wine, Semen Cassiae, and other dietary constituents (Walle, 2011). This compound exists in two isoforms *cis*- and *trans*-resveratrol, the isomer *trans* being more active than the *cis*-form (Amri et al., 2012). In preclinical studies, resveratrol has neuroprotective properties in both ischemic stroke, intracerebral hemorrhage (Bonsack et al., 2017; Zhao et al., 2019; Abd Aziz et al., 2020), subarachnoid hemorrhage (Zhao et al., 2017; Li and Han, 2018), and neurodegenerative disease (Griñán-Ferré et al., 2021). Resveratrol was reported to promote neurogenesis (Li et al., 2020) and reduce neurotoxicity by altering glial activity and signaling. In a randomized controlled trial, co-administration of resveratrol significantly improved the outcome of patients receiving delayed recombinant tissue plasminogen activator treatment (Chen et al., 2016). Subsequent preclinical studies have indicated that resveratrol treatment could reduce ischemic brain damage, yet there are some disputes over results. Some studies suggested that the low dosage of resveratrol was unable to induce a significant reduction (Pang et al., 2015; Faggi et al., 2018), and resveratrol administration without nanoparticles did not confer any neurological function recovery (Lu et al., 2020). Moreover, the administration dose, frequency, timing of treatment, and route in each study are so divergent that the overall therapeutic effect is difficult to evaluate. Treatment in some studies was a single dose of 100 mg/kg (He et al., 2017), while in other studies was a single dose of 20 mg/Kg (Teertam et al., 2020). To date, there is no meta-analysis available investigating the potential effects of resveratrol therapy in pre-clinical models of ischemic stroke. Addressing all these problems, we systematically assessed the bias of included studies and then summarized the optimal pattern of resveratrol therapy. This meta-analysis may provide significant clues and information for future clinical research.

## Materials and Methods

Preferred Reporting Items for Systematic Reviews and Meta-Analysis (PRISMA) was used to conduct this study (Moher et al., 2009). This meta-analysis was not registered in the International prospective register of systematic reviews (PROSPERO). However, the PROSPERO was carefully examined to make sure there is no registered meta-analysis that is investigating a similar topic.

### Search Strategy

Studies of resveratrol-based therapy for rodent models of cerebral ischemia were identified from PubMed, EMBASE, and Web of Science, from their inception to July 15, 2021, and using the following search strategy: (stroke OR cerebrovascular OR cerebral infarct OR cerebral ischemia/reperfusion OR middle cerebral artery OR middle cerebral artery occlusion) AND (resveratrol). The publication language was limited to English.

### Inclusion and Exclusion Criteria

The inclusion criteria were set up based on the PICOS-scheme (population, intervention, control, outcome, and study design). Published studies were included if they met the following criteria: 1) ischemic stroke animal model (rodent models); 2) testing the effects of purified resveratrol in at least one experimental group (no additional chemicals or drugs were used); 3) setting a control group with placebo; 4) providing adequate data on the functional outcome (neurobehavioral score measured on any scale/rotarod test) or the structural outcome (infarct volume) determined by a recognized method (such as TTC staining/Magnetic Resonance Imaging); 5) study: experimental studies presented in original research articles and 6) published in English.

The exclusion criteria were as follows: 1) animals treated with resveratrol analogues; 2) studies that only tested the effects of resveratrol combined with other chemicals or drugs (such as nanoparticles); 3) not reporting the number of animals in groups; 4) repeated publications or duplicate report, and abstracts without full text.

### Data Collection

The following information was abstracted by two investigators independently and discrepancies were resolved by consensus and then checked by a third investigator. 1) authors, year published, study country, 2) characteristics of the animals used, including species of animals, animal model, animal gender, anesthetic type, and animal number per group, 3) treatment information, including dosage, administration route, and timing, follow-up (the longest observation time of outcomes after occlusion), 4) the outcomes data: functional outcome (neurobehavioral score measured on any scale/rotarod test), structural outcome (infarction volume determined by TTC staining/Magnetic Resonance Imaging/cresyl violet staining/silver staining).

If a study comprised multi-experimental groups distinguished by dosage, frequency, delivery route, and timing that were compared with the control group, these experimental groups would be considered as independent comparisons. If the outcomes were evaluated at different follow-up times, only the longest follow-up time was collected. The GetData Graph Digitizer software was applied when only graphs were available.

### Quality Assessment

To evaluate the quality of the eligible studies, we used the Collaborative Approach to Meta-Analysis and Review of Animal Data from Experimental Studies (CAMARADES) checklists (Macleod et al., 2004). A sum of the quality scores was recorded for each study, with a total score of 10 points. Two researchers independently scored the included studies. Discrepancies were resolved by consensus and then adjudicated by a third investigator.

### Statistical Analysis

During data abstraction, we found that infarction volume determined by TTC staining (*n* = 46) and functional outcome determined by neurobehavioral score (*n* = 24) were available in large numbers of original studies. Thus, we decided to choose these as co-primary outcomes in this meta-analysis. Other secondary outcomes were rotarod test, and infarction volume determined by Magnetic Resonance Imaging/cresyl violet staining/silver staining. The combined effect size was calculated as standardized mean difference (SMD) with corresponding confidence interval (CI) between BMSCs treated group and control group. The random-effects model and Hedges calculation (Durlak, 2009) were used for the pooled SMD, and all analysis was performed with Stata 14.0 software. A *p* value <0.05 was considered statistically significant. The inconsistency index (*I* ^*2*^) was used to analyze heterogeneity (Higgins et al., 2003).

Four clinical characteristics were used to group the effect size of outcome: resveratrol dosage (<10, ≥10, ≤20, >20, and <50, 50–200 mg/Kg), frequency of treatment (single treatment; irregularly treatment; daily treatment), the timing of administration (pre-stroke onset; post-stroke onset), administration route (intraperitoneally; intravenously; oral gavage; intracarotid arterial). Subgroup analysis and meta-regression analysis (Higgins and Thompson, 2002) were conducted to explore the impact of the above clinical characteristics on outcomes and the possible sources of heterogeneity.

A leave-one-out sensitivity analysis was conducted by iteratively removing each study one by one to estimate the influence of each study.

Publication bias was evaluated by Egger’s tests, Trim and Fill analysis, and funnel plot (Egger et al., 1997; Vahidy et al., 2016). Plotting the SMD against the SE can cause distortion of funnel plots, especially when the included studies have small sample sizes. Thus, we plotted the SMD against $1/\sqrt{\text{n}}$ , a sample size-based precision estimate (Zwetsloot et al., 2017). Each funnel plot displays all studies in one plot with SMD as the x-value and $1/\sqrt{\text{n}}$ as the y-value.

## Results

### Study Selection

**FIGURE 1:**
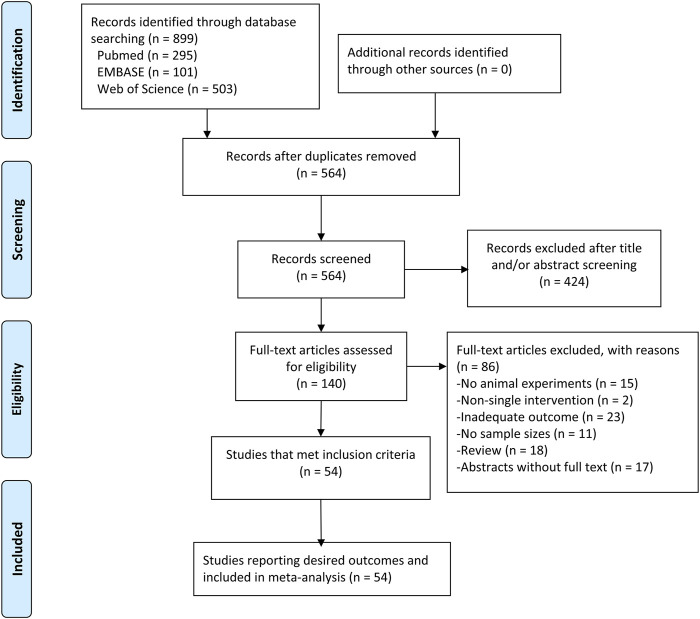
PRISMA flow diagram for review and selection process of studies included in meta-analysis of resveratrol in rodent models of ischemic stroke.

Electronic searching identified 295 articles in PubMed, 101 articles in EMBASE, and 503 articles in Web of Science. After removing duplicates, 564 articles were screened by abstract and/or title, resulting in 424 irrelevant records excluded. We retrieved the full text of the remaining 140 records for further assessment. Among them, 86 records were excluded due to review, abstracts without full text, not having purified resveratrol, no *in vivo* experiment, not reporting the number of animals in groups, and or no adequate outcomes (infarction volume or functional outcome determined by neurobehavioral score measured on any scale/rotarod test). Therefore, 54 studies (Huang et al., 2001; Sinha et al., 2002; Inoue, 2003; Gao et al., 2006; Tsai et al., 2007; Dong et al., 2008; Yousuf et al., 2009; Li et al., 2010; Sakata et al., 2010; Shin et al., 2010; Ren et al., 2011; Li et al., 2012; Shin et al., 2012; Hurtado et al., 2013; Lanzillotta et al., 2013; Lin et al., 2013; Orsu et al., 2013; Yan et al., 2013; Saleh et al., 2014; Wang et al., 2014; Fang et al., 2015; Hermann et al., 2015; Ishrat et al., 2015; Koronowski et al., 2015; Li et al., 2015; Narayanan et al., 2015; Pandey et al., 2015; Pang et al., 2015; Abdel-Aleem et al., 2016; Jeong et al., 2016; Li et al., 2016; Lopez et al., 2016; Su et al., 2016; Wan et al., 2016; Yang et al., 2016; Al Dera, 2017; He et al., 2017; Koronowski et al., 2017; Yu et al., 2017; Faggi et al., 2018; Hou et al., 2018; Liu et al., 2018; Dou et al., 2019; Grewal et al., 2019; Park et al., 2019; Yan et al., 2019; Alquisiras-Burgos et al., 2020; Lu et al., 2020; Mota et al., 2020; Pineda-Ramírez et al., 2020; Teertam et al., 2020; Yao et al., 2020; McDonald et al., 2021; Yu et al., 2021) met our criteria and were used for meta-analysis (Figure 1).

### Study Characteristics

The baseline characteristics of all studies are shown in Supplementary Tables S1, S2. All studies were carried out in rodents (rats and mice). The most common model of ischemic stroke was the t-MCAO induced with nylon monofilament, although other methods were also used, such as the photothrombosis, electrocoagulation, and embolic MCAO. The most common delivery route used for resveratrol was the intraperitoneal route. Others used were the intravenous, intracarotid arterial, and oral gavage routes. The dosage of resveratrol with intraperitoneal route ranged from 2.5 mg/kg to 100 mg/kg. Resveratrol was administrated either immediately after ischemic insult or over a period before ischemia onset. The follow-up time in most studies is 24 h. Infarction outcome was assessed by TTC staining in 46 studies, cresyl violet staining in four studies, silver-staining in one study, and MRI in one study. Behavioral outcomes were evaluated by behavioral scale (0 represents no neurological deficit) in 24 studies, rotarod test in four studies, limb function (beam walking test, limb-use asymmetry test, grip test, and gait assessment) in 5 studies, corner test in 2 studies, and Morris water maze test in one study. Considering that TTC staining and neurobehavioral score are the most common evaluations used in rodent studies of ischemic stroke, we took them as co-primary outcomes in this meta-analysis.

### Quality Assessment

The quality assessment of included studies is summarized in Table 1. The quality scores varied from 3 to 8, with a mean value of 5.94. All included studies were peer-reviewed publications. Most studies reported compliance with animal welfare regulations. However, only one study was performed on aged animals (20-month-old aged mice) (Jeong et al., 2016), no study reported a sample size calculation. Control of temperature was stated in 40 studies. 38 studies reported random allocation to treatment or control, 31 studies reported blinding assessment of outcome, 23 studies stated blinded induction of model, and 32 studies declared no potential conflict of interests. The details of the quality assessment are presented in Supplementary Table S3.

**TABLE 1:** Percentage of included studies satisfying each criterion of CAMARADES checklists.

Quality score criterion	Percentage of qualified studies (%)
Publication in a peer-reviewed journal	100
Control of temperature	74.07
Randomized treatment allocation	70.37
Allocation concealment	42.59
Use of aged animal models	1.85
Blind assessment of outcome	57.41
Avoidance neuroprotective anesthetics	94.44
Sample size calculation	0
Compliance with animal welfare regulations	92.59
Statement of conflict of interest	59.25

### Meta-Analysis

**FIGURE 2:**
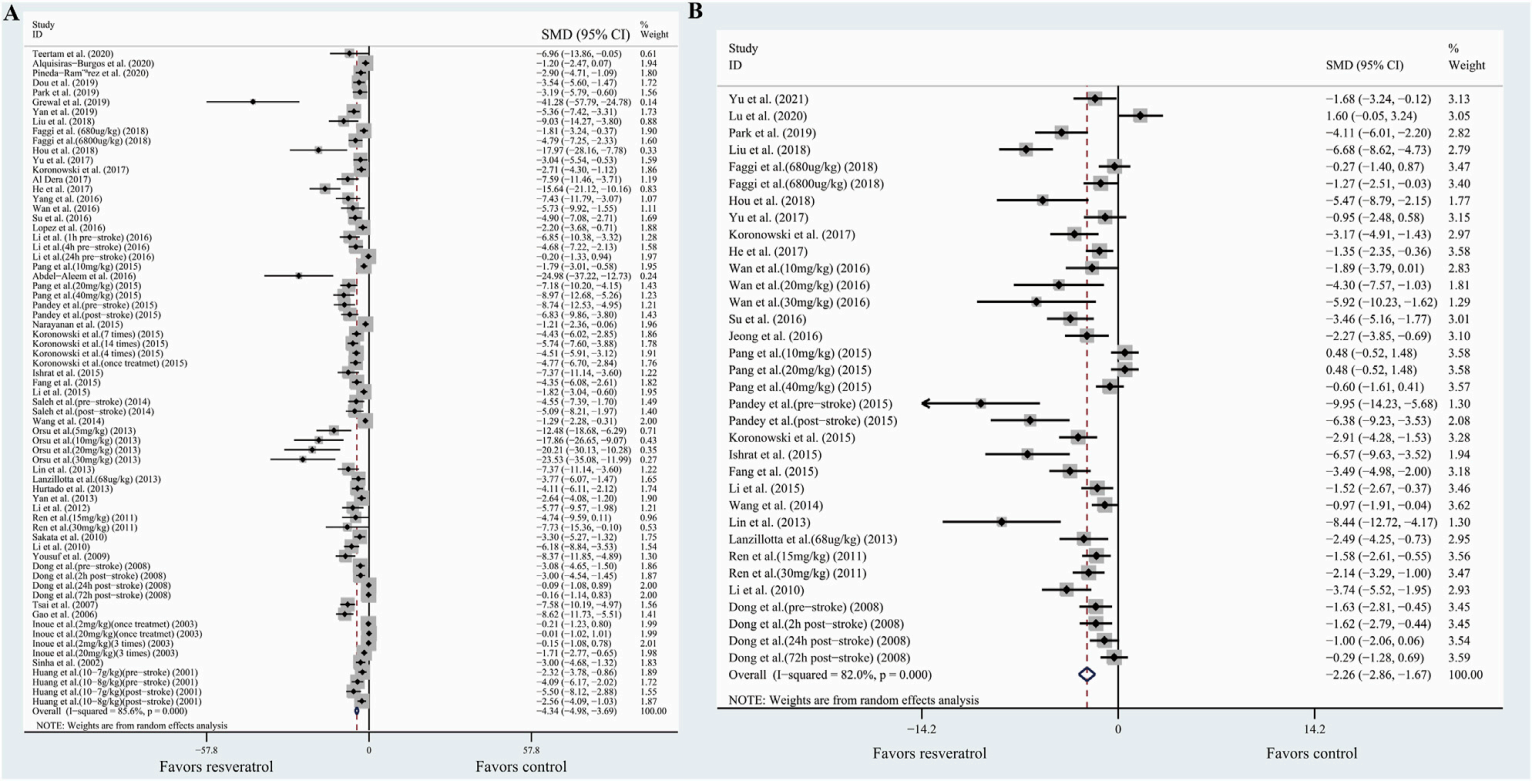
Forest plot shows mean effect size and 95% CI for **(A)** TTC staining, **(B)** neurobehavioral score between resveratrol therapy group and control group. Weights have been calculated using random effects model. Degree of heterogeneity in the pooled estimates is represented at *I* ^*2*^ statistic. Abbreviations: SMD, standardized mean difference; CI: confidence interval.

Our primary aim was to evaluate whether resveratrol had neuroprotective effects on ischemic stroke. The primary outcome was composed of two aspects: infarction volume determined by TTC staining, and behavioral outcomes determined by neurobehavioral score. Meta-analysis of 46 studies with 68 comparisons showed significant effects of resveratrol for reducing infarct volume compared with control groups (SMD −4.34; 95% CI −4.98 to −3.69; *p* < 0.001; I^2^ = 85.6%; Figure 2A).

Meta-analysis of 24 studies with 34 comparisons reported the neurobehavioral score. The pooled analysis showed that resveratrol can significantly improve the neurological function compared with the control groups (SMD −2.26; 95% CI −2.86 to −1.67; *p* < 0.001; *I* ^2^ = 82.0%; Figure 2B).

We also conducted pooled analysis for the secondary outcomes: infarction outcome assessed by cresyl violet-staining/silver-staining/MRI (*n* = 10), and behavioral outcomes evaluated by rotarod test (*n* = 5). The result was similar: The composite weighted mean (95% CI) effect size for rotarod tests was 2.59 (0.74, 4.44) (*p* <0 .001, *I* ^*2*^ = 91.9%), and −1.63 (−2.68, −0.58) (*p* <0 .0011, *I* ^*2*^ = 87.0%) for infarction outcome assessed by cresyl violet-staining/silver-staining/MRI. (Supplementary Figures S1A,B).

### Stratified Analysis

To identify heterogeneity potentially influencing the analysis, articles were divided into several groups based on dosage, frequency of treatment, the timing of administration, and administration route. Table 2 summarizes the data of primary outcomes in diverse subgroup analysis. Due to the insufficient number of comparisons, stratified analysis for rotarod test and infarction outcome assessed by cresyl violet-staining/silver-staining/MRI were not conducted.

**TABLE 2:** Subgroup analysis of primary outcomes (TTC staining and neurobehavioral score) in animal models of ischemic stroke associated with resveratrol therapy.

Variable	TTC staining						Neurobehavioral score					
	No. of reports	Pooled estimates (95% CI)	Q statistic	*p* Value for heterogeneity	*I* ^*2*^ value (%)	Between group *p* value	No. of reports	Pooled estimates (95% CI)	Q statistic	*p* Value for heterogeneity	*I* ^*2*^ value (%)	Between group *p* value
Dosage						0.044						0.002
<10	18	−3.76 (−4.88, −2.64)	107.68	<0.001	84.2%		5	−2.15 (−3.63, −0.67)	17.33	<0.001	76.9%	
≥10 and ≤20	23	−3.93 (−4.92, −2.94)	129.49	<0.001	83.0%		9	−1.22 (−2.32, −0.12)	46.26	<0.001	82.7%	
>20 and <50	16	−6.02 (−7.92, −4.13)	118.37	<0.001	87.3%		12	−3.10 (−4.20, −1.99)	55.82	<0.001	80.3%	
50–200	11	−4.72 (−6.55, −2.90)	100.84	<0.001	90.1%		8	−2.48 (−3.73, −1.23)	48.73	<0.001	85.6%	
Frequency						0.001						0.09
Once	34	−4.32 (−5.27, −3.37)	230.36	<0.001	85.7%		11	−2.59 (−3.80, −1.37)	63.47	<0.001	84.2%	
Daily	29	−4.31 (−5.30, −3.32)	199.26	<0.001	85.9%		21	−2.01 (−2.71, −1.31)	105.36	<0.001	81.0%	
Unregularly	5	−4.79 (−6.77, −2.81)	21.73	<0.001	81.6%		2	−3.83 (−8.77,1.09)	9.2	<0.001	89.1%	
Administration timing						0.32						0.731
Pre	42	−4.02 (−4.78, −3.27)	266.72	<0.001	84.6%		21	−2.43 (−3.23, −1.64)	115.54	<0.001	82.7%	
Post	26	−5.03 (−6.26, −3.79)	196.77	<0.001	87.3%		13	−2.03 (−2.97, −1.11)	67.2	<0.001	82.1%	
Administration route						<0.001						0.001
Intraperitoneal	43	−5.14 (−6.01, −4.27)	232.99	<0.001	82.0%		24	−2.89 (−3.59, −2.20)	99.91	<0.001	77.0%	
Intravenous	10	−4.07 (−5.38, −2.75)	36.87	<0.001	75.6%		1	NA	NA	NA	NA	
Oral	15	−2.52 (−3.57, −1.47)	111.83	<0.001	87.5%		8	−0.71 (−1.37, −0.06)	20.04	0.001	65.1%	
Intraarterial	NA	NA	NA	NA	NA		1	NA	NA	NA	NA	

For TTC staining, no significant between-subgroup heterogeneity was found in administration timing (*p* = 0.32). Significant differences between-subgroup were found in the dosage (*p* = 0.044), and frequency of administration (*p* < 0.001), and administration route (*p* < 0.001). Among them, there was a clear difference in therapeutic effect by the dosage of resveratrol. Compared with −3.93 (95% CI, −4.92 to −2.94) for doses between 10 and 20 mg/kg and −3.76 (95% CI, −4.88 to −2.64) for doses less than 10 mg/kg, the effects size for doses between 20 and 50 mg/kg is −6.02 (95% CI, −7.92 to −4.13) (Table 2).

For the neurobehavioral score, no significant between-subgroup heterogeneity was found in administration timing (*p* = 0.731) and frequency of administration (*p* = 0.09). Significant differences between-subgroup were found in the dosage (*p* = 0.002), and administration route (*p* = 0.001). Similarly, there was a significant difference in treatment effect by dosage of resveratrol. Compared with −1.22 (95% CI, −2.32 to −0.12) for doses between 10 and 20 mg/kg and −2.15 (95% CI, −3.63 to −0.67) for doses less than 10 mg/kg, the effects size for doses between 20 and 50 mg/kg is −3.10 (95% CI, −4.20 to −1.99) (Table 2). Thus, we speculated that resveratrol treatment with 20–50 mg/kg achieves the greatest effects.

**FIGURE 3:**
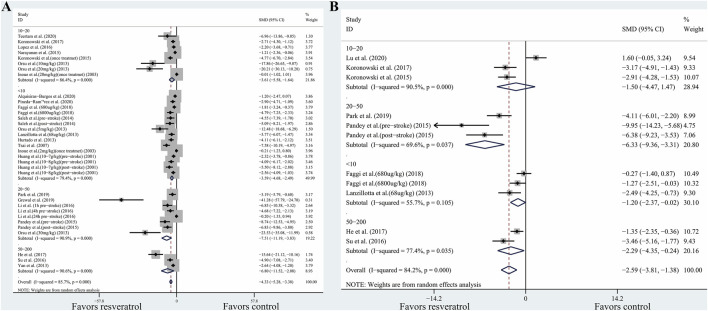
**(A)** Effect of resveratrol dose on TTC staining (only including comparisons with single treatment). **(B)** Effect of resveratrol dose on neurobehavioral score (only including comparisons with single treatment). Abbreviations: SMD, standardized mean difference; CI: confidence interval.

However, in the included studies, resveratrol dosage was confounded with other variables. For instance, out of the 18 comparisons involving lower doses (<10 mg/kg), 15 of them administered the resveratrol with a single treatment instead of daily treatment. This makes it difficult to identify whether the difference in treatment effect was related to the dosage or the frequency of administration. To elucidate the effect of dose independently from administration frequency, we assessed the dosage effect for comparisons only involving single treatment. In the comparisons involving a single treatment, the estimated effect of the 20–50 mg/kg dose in this subset was similar to the full analysis (Figures 3A,B). This implied that the smaller effect estimated in the lower dose (<10 mg/kg) are indeed associated with the lower dose instead of administration frequency.

Except for the administration frequency, the routes of administration may be correlated to the different effects in dosage. In the 16 comparisons involving 20–50 mg/kg dose, 15 of them administered the resveratrol with the intraperitoneal route. To elucidate the effect of dosage independently from administration routes, we assessed the dosage effect for comparisons only involving the intraperitoneal route. For TTC staining, significant differences between-subgroup were found (*p* < 0.001). Compared with −4.219 (95% CI, −5.282 to −3.156) for dosage between 10 and 20 mg/kg and −4.23 (95% CI, −6.26 to −2.211) for dosage less than 10 mg/kg, the effects size for dosage between 20 and 50 mg/kg is −5.754 (95% CI, −7.666 to −3.843). This implied that the smaller effect estimated in the lower dose (<20 mg/kg) is indeed associated with the lower dose instead of administration routes. Similar results were also found in the outcomes of the neurobehavioral scores. However, in the comparisons only involving the intraperitoneal route, the dosage between 50 and 200 mg/kg achieved the greatest effects size (SMD, −8.35; 95% CI, −11.63 to −5.07), which is different from the full analysis. We speculated that the difference may owe to the administration routes. In the full analysis, some studies using dosage between 50 and 200 mg/kg delivered the resveratrol orally. The bioavailability of the oral route is less than the intraperitoneal route. Thus, the larger effects estimated in the higher dose (>50 mg/kg) may associate with the administration routes.

### Meta-Regression Analysis

For infarct volume, we discovered that administration timing (*p* = 0.448), frequency (*p* = 0.787), and dosage (*p* = 0.288) had no significant relation with heterogeneity, only delivery route presented significantly related with the reduction of infarction volume (*p* = 0.033). Similarly, for neurobehavioral score, delivery route (*p* = 0.002) was a significant source of heterogeneity, while administration timing (*p* = 0.962), frequency (*p* = 0.726), and dosage (*p* = 0.188) had little effect on heterogeneity (Table 3). Thus, the impact of the delivery route on the outcome is the possible source of high heterogeneity.

**TABLE 3:** Meta-regression analysis.

Covariates	TTC staining			Neurobehavioral score		
	Coefficient	95% CI	*p* Value	Coefficient	95% CI	*p* Value
Dosage	−0.493915	−1.414301; 0.427471	0.288	−0.470948	−1.186008; 0.244111	0.188
Frequency	−0.213211	−1.78268; 1.356259	0.787	−0.258621	−1.755727; 1.238486	0.726
Timing	−0.781463	−2.828497; 1.265571	0.448	0.038947	−1.619807; 1.697703	0.962
Route	1.215059	0.099148; 2.33097	0.033	1.227027	0.470639; 1.983415	0.002

### Sensitivity Analysis and Publication Bias

To assess the robustness of the estimated pooled analysis for infarction volume and neurobehavioral score, we used a leave-one-out sensitivity analysis by systematically removing each study and recalculating the pooled effect size of the remaining studies. For TTC staining and neurobehavioral score, the pooled effect was stable, which indicates that the results were not driven by any single study.

**FIGURE 4:**
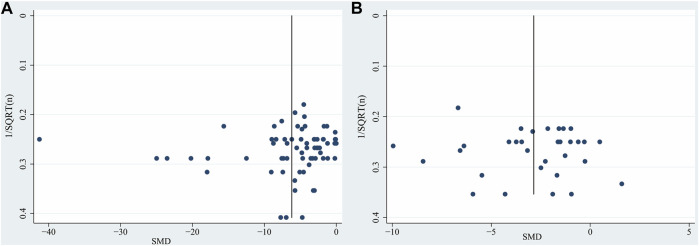
Funnel plot for **(A)** TTC staining, **(B)** neurobehavioral score. Each funnel plot displays all studies in one plot with SMD as the x-value and 1/√n as the y-value. Abbreviations: SMD, standardized mean difference.

The publication bias was evaluated by funnel plots and Egger’s regression test. It has been demonstrated that the use of SMD to assess publication bias can lead to distortion of results due to over-estimation (Zwetsloot et al., 2017). For this reason, the funnel plot is a graphical representation of trial size plotted against the reported effect size. Inspection of the funnel plots revealed slight asymmetry for TTC staining and neurobehavioral score (Figures 4A,B). In addition, we performed Egger’s test, which indicated that no significant publication bias for TTC staining (*p* = 0.480) and neurobehavioral score (*p* = 0.691).

## Discussion

To our knowledge, this is the first preclinical meta-analysis to investigate the neuroprotective effect of resveratrol treatment in animals subjected to ischemic stroke.

### Summary of Evidence

The following is a summary of these results: 1) Resveratrol has neuroprotective effects in alleviating infarct volume and ameliorating neurobehavioral defects in rodent models of ischemic stroke. 2) The dose of resveratrol was correlated with effect size in TTC staining and neurobehavioral score. 20–50 mg/kg resveratrol therapy showed the greatest efficacy. 3) Compared with the administration of resveratrol intravenous and oral, intraperitoneal treatment presented more effective to reduce infarction volume. However, in clinical application, the intravenous and oral route is more common. The subgroup analysis in our meta-analysis suggested that intravenous treatment achieved greater efficacy than oral treatment, possibly due to the increased bioavailability with intravenous treatment. 4) There were no significant differences between the estimated pooled effect size for a single treatment and daily treatment. 5) The administration timing of resveratrol in our included studies ranges from 30-days before ischemia onset to 3-days after ischemia onset. The neuroprotection between the pre-stroke treatment sub-group and post-stroke treatment sub-group was not significant, which suggested that resveratrol has a relatively long therapeutic time window.

### Pharmacokinetics and Pharmacodynamics Properties of Resveratrol

The pharmacokinetics and pharmacodynamics properties of resveratrol have been studied in several studies. Due to resveratrol’s low water solubility (<50 μg/ml) and high permeability, it is classified as the second class of the biopharmaceutical classification system (Singh and Pai, 2015). The principal absorption site is at the intestine through passive diffusion or forming complexes with membrane transporters (Sergides et al., 2016). Resveratrol can be absorbed through the bloodstream to the liver, where it is metabolized to form glucuronide, and sulphate derivatives or free. The free form can be bound in a non-covalent manner to proteins, such as albumin and lipoproteins (Burkon and Somoza, 2008). These complexes can be dissociated at cellular membranes that have receptors for albumin and lipoproteins, leaving the resveratrol free and allowing it to enter cells. The peak plasma concentration in humans was reached at 90 min with a single oral dose treatment of 25 mg. The half-life time of plasma concentration is around 9.2 h (Walle et al., 2004). Owing to its lipophilic characteristics, resveratrol has high absorption (at least 70% after oral consumption), and a high volume of distribution supporting its potential to accumulate in tissues such as the brain. Although resveratrol has a high absorption rate (Walle et al., 2004), the rapid metabolism of resveratrol leads to approximately 1% bioavailability of the parent compound (Walle, 2011). Except for the low solubility and high metabolism, an additional specific problem for the delivery of appropriate therapeutic resveratrol concentrations in the brain tissues is the presence of the blood-brain barrier. Peripheral administration of resveratrol could increase the antioxidant enzyme activities in the brain of healthy rats, which suggested that resveratrol is able to traverse the blood-brain barrier, and have biological activity in the brain (Mokni et al., 2007). A previous study suggested that only 2% of plasmatic resveratrol can cross the blood-brain barrier (Asensi et al., 2002). Despite its low bioavailability, resveratrol presents significant efficacy in the brain tissues, and which may ascribe to the metabolites (Walle et al., 2004). The metabolites of resveratrol, such as resveratrol-3-*O*-glucuronide, resveratrol-*O*-glucuronide, resveratrol-3-*O*-sulfate, and resveratrol-4′-*O*-sulfate, possess anti-inflammatory and antioxidant properties (Luca et al., 2020). A previous study reviewed the neuroprotection provided by resveratrol in brain tissues of animals, such as preserving mitochondrial function, inhibiting the lipid peroxidation, and inducing phosphorylation of several mitogen activated protein kinases (Shetty, 2011). Despite the ability of resveratrol to cross the blood-brain barrier, recent research aims to explore the methods improving the permeability and stability of resveratrol in the central nervous system. Nanotechnology has been proposed for the incorporation of resveratrol-loaded nanocarriers designed to deliver resveratrol to brain tissues (Fonseca-Santos and Chorilli, 2020). The nanocarriers containing resveratrol reduced infarct volume and improved neurobehavioral outcomes after ischemic stroke in rats (Ashafaq et al., 2021).

As dietary polyphenolic phytoalexin, resveratrol appeared to be well tolerated, and non-toxic. In an experimental study, resveratrol did not cause any adverse effects in rats at 28 daily doses of 50, 150, or 500 mg/kg (Williams et al., 2009). In a clinical trial conducted in healthy volunteers, resveratrol was demonstrated to be safe with 29 daily doses of 0.5, 1.0, 2.5, and 5.0 g, except the 2.5 and 5.0 g doses caused gastrointestinal symptoms, including nausea, flatulence, abdominal discomfort, and diarrhea (Brown et al., 2010).

### Possible Mechanism of Resveratrol-Mediated Neuroprotection

**FIGURE 5:**
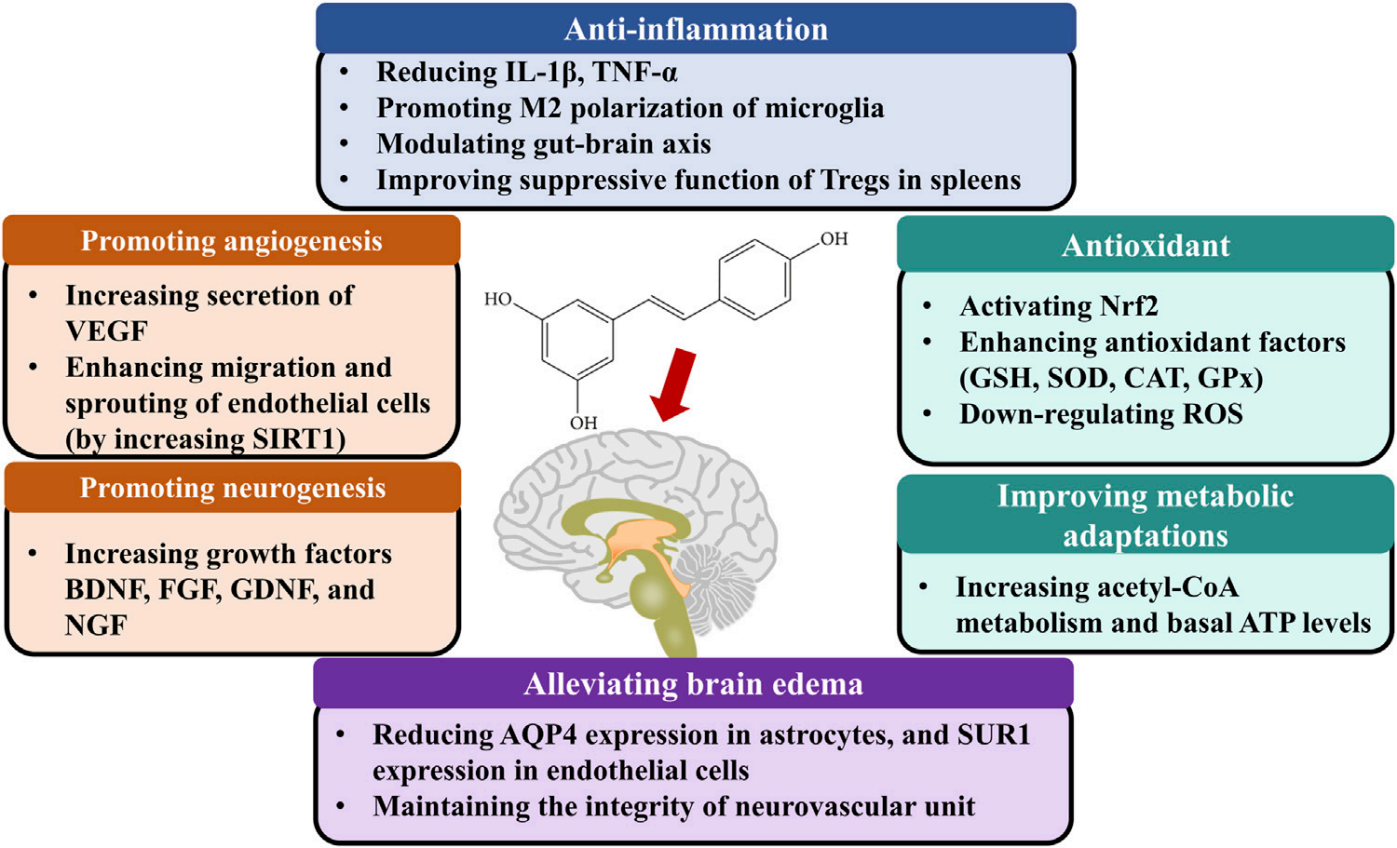
The possible mechanisms of resveratrol therapy for ischemic stroke. Abbreviations: BDNF, Brain-derived neurotrophic factor; CAT, Catalase; EGF, Epidermal growth factor; FGF, Fibroblast growth factor; NGF, Nerve growth factor; Nrf2, Transcription factor nuclear factor (erythroid-derived 2)-like 2; GDNF, Glial cell line-derived neurotrophic factor; Glial cell-derived neurotrophic factor; GPx, glutathione peroxidase; GSH, Glutathione; Interleukin 1β, IL-1β; SOD, Superoxide dismutase; SIRT1, Silent mating type information regulation 2 homolog 1; TNF-α, Tumor necrosis factor-alpha; VEGF, Vascular endothelial growth factor.

The studies included in our meta-analysis indicated the main mechanisms of neuroprotection include the following biological activities (Figure 5): 1) Promoting angiogenesis. In an *in vitro* study, resveratrol-induced endothelial nitric oxide synthase phosphorylation led to prompt generation of nitric oxide in endothelial cells. The elevated nitric oxide increased the secretion of VEGF and matrix metalloproteinases (MMPs) (Simão et al., 2012). *In vivo* model, resveratrol administration elevated matrix metalloproteinase-2 and vascular endothelial growth factor levels (Dong et al., 2008). Moreover, resveratrol is an activator of silent information regulator 2 homologue 1, which enhances angiogenesis through migration, and sprouting of endothelial cells (Koronowski et al., 2017). 2) Promoting neurogenesis. Resveratrol treatment significantly increased the expression rates of neuronal markers with bromodeoxyuridine in the ischemic lesion site (Hermann et al., 2015). 3) Inhibiting neuroinflammation. Resveratrol reduced interleukin-1β, tumor necrosis factor-α protein levels, and immunoglobulin G extravasation in the brain tissues (Jeong et al., 2016). Meanwhile, Resveratrol promoted the M2 polarization of microglia after cerebral ischemia (Ma et al., 2020). In addition, resveratrol modulated inflammation by targeting the gut-brain axis, such as regulating Th17/Tregs and Th1/Th2 polarity shift in the small intestinal lamina propria (Dou et al., 2019). Resveratrol pretreatment also improved the suppressive function of Tregs in the spleens, which increased levels of anti-inflammatory factors, and decreased levels of pro-inflammatory factors in the plasma and ischemic hemisphere (Yang et al., 2016). 4) Antioxidant. Oxidative stress plays a pivotal role in neurological dysfunction. Resveratrol delayed the increases in oxygen species in brain tissue after ischemia, decreased xanthine oxidase activity and expression levels of inducible nitric oxide synthase, and increased levels of antioxidant enzymes such as superoxide dismutase, glutathione peroxidase, and chloramphenicol acetyltransferase (Su et al., 2016; Al Dera, 2017; Alquisiras-Burgos et al., 2020). 5) Improving metabolic adaptations. Brain tissues may lack metabolic plasticity due to their tight regulation of energy metabolism (Khoury et al., 2016). Compared with the control group, the cortex with resveratrol preconditioning presented increasing acetyl-CoA metabolism, basal ATP levels, and long-term ischemic tolerance (Khoury et al., 2019). 6) Alleviating brain edema. Astrocytic swelling mediated by AQP4 plays a significant role in cytotoxic edema. Sulfonylurea receptor 1 (SUR1) interacted with AQP4 to form a heteromultimeric complex favoring ion/water osmotic coupling and cell swelling. Following brain injury, SUR1 is up-regulated in the cells from the neurovascular unit. Resveratrol was demonstrated to reduce AQP4 expression (Li et al., 2015; Alquisiras-Burgos et al., 2020) in astrocytes, and SUR1 expression in endothelial cells (Alquisiras-Burgos et al., 2020) after ischemic stroke. Except for the endothelial cell and astrocyte, the interconnections between cells also contribute to brain edema. The neurovascular unit is a physiological and functional unit encompassing human brain microvascular endothelial cells, pericytes, smooth muscle cells, astrocytes, microglia, and neurons. The integrity of the neurovascular unit may determine the evolution of blood-brain barrier damage, neuronal death, and neuroinflammation. MMP-9 has been shown to degrade components of the basal lamina matrix. Some studies found that resveratrol could inhibit MMP-2 and MMP-9 activity in human cerebral microvascular endothelial cells (Cavdar et al., 2012; Pandey et al., 2015; Wei et al., 2015), which maintain the integrity of the neurovascular unit and decrease BBB permeability. However, how resveratrol regulates cell-cell signaling in the neurovascular unit remains further studied.

### Limitations

There are several limitations in terms of drawing definitive conclusions. 1) our study only included published data in English, which may lead to a certain degree of selective bias. 2) we limited outcomes measures in infarct volume and neurobehavioral score. Thus, we may disregard results seen in other outcomes. 3) the follow-up time in most included studies is 24 h, few studies evaluated the outcomes on 28 days post-stroke. Thus, it remains further research whether resveratrol plays an effective long-term treatment therapy for ischemic stroke.

### Clinical Application

Some previous treatments that have shown great efficacy in animal studies have failed to apply in humans, possibly owing to the side effects, and narrow therapeutic time windows (Mergenthaler and Meisel, 2012). The present preclinical meta-analysis suggested that resveratrol has a relatively long therapeutic time window in the animal model. The administration timing of resveratrol in our included studies ranges from 30-days before ischemia onset to 3-days after ischemia onset. However, there is still significant work to be done for clinical application. First, age is one of the non-modifiable risk factors of ischemic stroke (Campbell and Khatri, 2020). Nevertheless, the included studies are based almost exclusively on healthy adult animals. It is doubtful whether resveratrol can achieve the same effect in the elderly animal models. In addition, no studies in the present meta-analysis evaluated the potential side effects of resveratrol injection on ischemic stroke. Resveratrol, when administered at a high dose (1,000 mg/kg/day), may cause renal and hepatic toxicity (Crowell et al., 2004; Rocha et al., 2009). We are incapable of evaluating the safety of resveratrol treatment from the meta-analysis. However, a previous clinical study suggested that resveratrol 2000 mg twice daily was well tolerated by healthy subjects (la Porte et al., 2010). Thus, the translation of resveratrol for the therapy of ischemic stroke is promising.

## Conclusion

Based on the data of this meta-analysis, resveratrol treatment presents neuroprotection compared with control groups, by assessing the treatment outcomes including infarct volume, and neurobehavioral score. Furthermore, we suggested that the dosage ranging from 20 to 50 mg/kg showed the greatest efficacy. The results of this meta-analysis may provide certain references and a baseline for further preclinical and clinical studies with important implications for human health.

## Data Availability

The original contributions presented in the study are included in the article/Supplementary Material, further inquiries can be directed to the corresponding author.

## Supplementary Material

Supplementary Figure 1 Forest plot shows mean effect size and 95 % CI for **(A)** infarction outcome assessed by cresyl violet-staining/silver-staining/MRI, **(B)** rotarod tests between resveratrol therapy group and control group. Weights have been calculated using random effects model. Degree of heterogeneity in the pooled estimates is represented at I2 statistic. Abbreviations: SMD, standardized mean difference; CI: confidence interval.
