# Supplementary figures and images for: Resveratrol has an Overall Neuroprotective Role in Ischemic Stroke: A Meta-Analysis in Rodents
Source: Front Pharmacol. 2021 Dec 20;12:795409. doi: 10.3389/fphar.2021.795409 (PMC8721173; doi:10.3389/fphar.2021.795409)

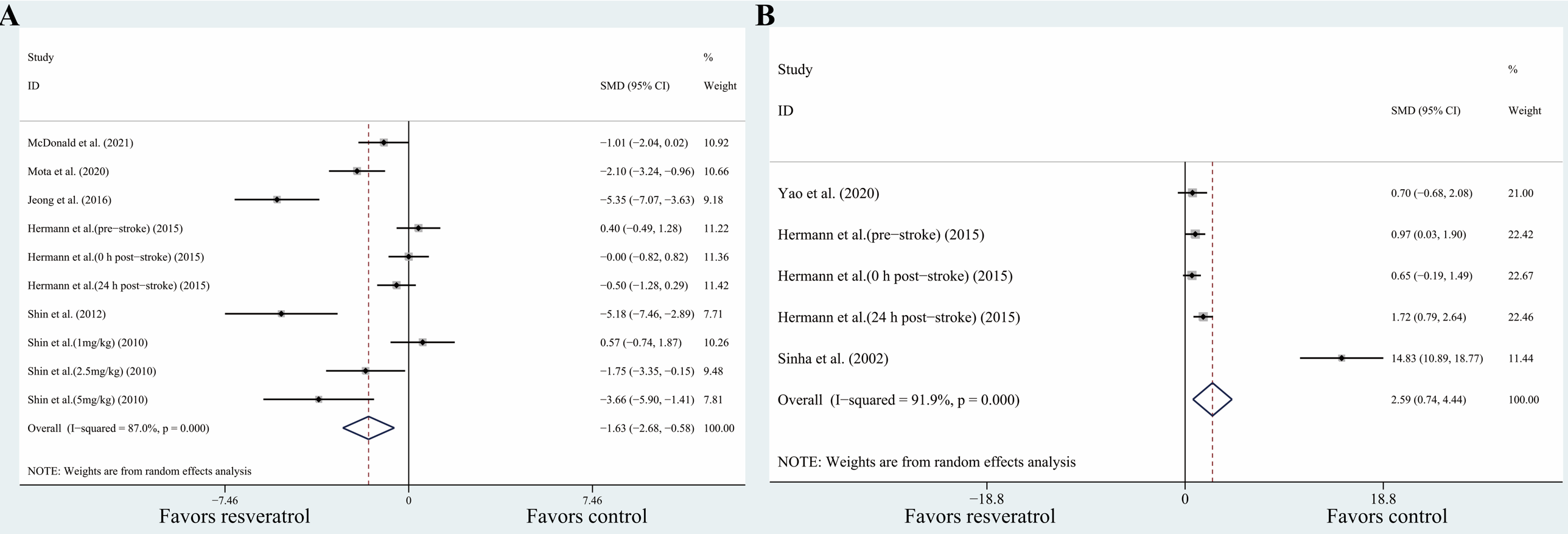

Supplement: Supplementary file 1 [file Image1.TIF]
