# Supplementary material for: Resveratrol has an Overall Neuroprotective Role in Ischemic Stroke: A Meta-Analysis in Rodents
Source: Front Pharmacol. 2021 Dec 20;12:795409. doi: 10.3389/fphar.2021.795409 (PMC8721173; doi:10.3389/fphar.2021.795409)
Supplement: Supplementary file 2 [file DataSheet1.docx]

**Supplementary Tables**

**Supplementary Table 1. Characteristics of the studies included for the meta-analysis.**

| Sl.no | Reference | Animals | Anesthetic | Model | Dosage | Timing | Follow-up | Vehicle | Approach |
| --- | --- | --- | --- | --- | --- | --- | --- | --- | --- |
| 1 | (Yu et al., 2021) | Rats, male | NA | Filament; t-MCAO | 30 mg/kg; daily; 7 days | 7 d before ischemia onset | 14 days | ethyl ethanol | ip |
| 2 | (McDonald et al., 2021) | Rats, female | Isoflurane | Photothrombosis; | 5 mg/kg; daily; 3 weeks | 24 h after ischemia onset | 72 hours/ 6 weeks | β-cyclodextrin | ip |
| 3 | (Yao et al., 2020) | Rats, male | Pentobarbitalsodium | Photothrombosis; Sensory cortex | 10 mg/kg; daily; 4 weeks | 7 d after ischemia onset | 5 weeks | NA | ip |
| 4 | (Teertam et al., 2020) | Rats, male | Ketamine; Xylazine | Filament; t-MCAO | 20 mg/kg; single | 30 min before ischemia onset | 24 hours | NA | ip |
| 5 | (Mota et al., 2020) | Mice, male | Tribromoethanol | Electrocoagulation; pMCAO | 680 μg/kg; single | Immediately after ischemia onset | 2 days | NA | ip |
| 6 | (Lu et al., 2020) | Rats, male | Isoflurane | Filament; t-MCAO | 10 mg/kg;  single | Immediately after ischemia onset | 24 hours | Saline | intracarotid arterial |
| 7 | (Alquisiras-Burgos et al., 2020) | Rats, male | Isoflurane | Filament; t-MCAO | 1.9 mg/kg;  single | Immediately after ischemia onset | 24 hours | ethanol | iv |
| 8 | (Pineda-Ramírez et al., 2020) | Rats, male | Isoflurane | Filament; t-MCAO | 1.8 mg/Kg; single | Immediately after ischemia onset | 24 hours | ethanol | iv |
| 9 | (Dou et al., 2019) | Mice, male | Ketamine; Xylazine | Filament; t-MCAO | 10 mg/kg;  daily; 3 days | Immediately after ischemia onset | 3 days | DMSO | ip |
| 10 | (Park et al., 2019) | Rats, male | Zoletil | Filament; t-MCAO | 30 mg/kg; single | Immediately after ischemia onset | 24 hours | DMSO | ip |
| 11 | (Grewal et al., 2019) | Mice, male | Chloral hydrate | BCCA | 30 mg/kg; single | after ischemia onset | 24 hours | NA | ip |
| 12 | (Yan et al., 2019) | Rats, male | pentobarbital sodium | BCCA | 20 mg/kg; daily; 7 days | 7 d before ischemia onset | NA | Saline | ip |
| 13 | (Liu et al., 2018) | Rats, male | Chloral hydrate | Filament; t-MCAO | 50 mg/kg; daily; 7 days | 7 d before ischemia onset | 24 hours | DMSO | ip |
| 14 | (Faggi et al., 2018) | Mice; male | NA | Filament; t-MCAO | 680/6800 μg/kg; single | 30 min after ischemia onset | 24 hours | NA | ip |
| 15 | (Hou et al., 2018) | Rats, male | chloral  hydrate | Filament; t-MCAO | 30 mg/kg; daily; 7 days | 7 d before ischemia onset | 24 hours | DMSO | ip |
| 16 | (Yu et al., 2017) | Rats, male | Chloral hydrate | Filament; t-MCAO | 30 mg/kg; daily; 7 days | 7 d before ischemia onset | 24 hours | ethyl ethanol | ip |
| 17 | (Koronowski et al., 2017) | Mice, male | Isoflurane | Filament; t-MCAO | 10 mg/kg; single | 2 d before ischemia onset | 24 hours | DMSO | ip |
| 18 | (Al Dera, 2017) | Rats, male | Chloral hydrate | BCCA | 20 mg/kg; daily; 30 days | 30 d before ischemia onset | NA | hydroxypropylcyclodextrin | ip |
| 19 | (He et al., 2017) | Rats, male | Chloral hydrate | Filament; t-MCAO | 100 mg/kg; single | Immediately after ischemia onset | 24 hours | DMSO | ip |
| 20 | (Yang et al., 2016) | Rats, male | Chloral hydrate | Filament; t-MCAO | 50 mg/kg; daily; 7 days | 7 d before ischemia onset | 24 hours | DMSO | ip |
| 21 | (Wan et al., 2016) | Rats, male | NA | Filament; t-MCAO | 10/20/30 mg/kg; daily; 5 days | 5 d before ischemia onset | 24 hours | DMSO | ip |
| 22 | (Su et al., 2016) | Rats, male | Enflurane | Filament; t-MCAO | 100 mg/kg; single | 10 min after reperfusion | 48 hours/14 days | ethanol | ip |
| 23 | (Lopez et al., 2016) | Mice, male | Isoflurane | Filament; t-MCAO | 10 mg/kg; single | 3 d before ischemia onset | 72 hours | saline | ip |
| 24 | (Li et al., 2016) | Rats, male | Chloral hydrate | Filament; t-MCAO | 30 mg/kg; single | 1/4/6/12/24 h before ischemia | 24 hours | saline | ip |
| 25 | (Jeong et al., 2016) | Mice,  female | Isoflurane | Filament; t-MCAO | 0.1 mg/kg; daily; 10 days | 7 d before ischemia | 72 hours | ethanol | oral gavage |
| 26 | (Abdel-Aleem et al., 2016) | Rats, male | sodium pentobarbital; ketamine | Filament; t-MCAO | 20 mg/kg; daily; 30 days | 30 d before ischemia onset | 24 hours | saline | oral gavage |
| 27 | (Pang et al., 2015) | Rats, male | Isoflurane | Filament; t-MCAO | 10/20/40 mg/kg; daily; 7 days | 7 d before ischemia onset | 22 hours/ 14 days | NA | oral gavage |
| 28 | (Pandey et al., 2015) | Rats, male | Ketamine; Xylazine | Filament; t-MCAO | 40 mg/kg; single | 30min before/2h after ischemia onset | 24 hours | NA | ip |
| 29 | (Narayanan et al., 2015) | Mice, male | NA | Filament; t-MCAO | 10 mg/kg; single | 2 d before ischemia onset | 24 hours | DMSO | ip |
| 30 | (Koronowski et al., 2015) | Mice, male | Isoflurane | Filament; t-MCAO | 10 mg/kg; daily/ every other day/single/intermittent; 14 days | 14 d before ischemia onset | 24 hours | DMSO | ip |
| 31 | (Ishrat et al., 2015) | Mice, NA | Isoflurane | Embolic MCAO | 5 mg/kg; twice | Immediately and 3 h after ischemia onset | 24 hours | NA | iv and ip |
| 32 | (Hermann et al., 2015) | Mice; male | Isoflurane | Filament; t-MCAO | 50 mg/kg; daily | Prophylactic/acute/post-acute | 28 days | DMSO | ip |
| 33 | (Fang et al., 2015) | Rats, male | Chloral hydrate | Filament; t-MCAO | 30 mg/kg; daily; 4 days | 3 h after ischemia onset | 24 hours | DMSO | ip |
| 34 | (Li et al., 2015) | Rats, male | Chloral hydrate | Filament; t-MCAO | 20 mg/kg; three times | 10 min pre and 0 h, 20h post ischemia | 24 hours | NA | ip |
| 35 | (Saleh et al., 2014) | Rats, male | sodium thiobutabarbital | Filament; t-MCAO | 2x10^-3^ mg/kg; single | 30 min before/after ischemia onset | 24 hours | propylene glycol | iv |
| 36 | (Wang et al., 2014) | Rats, male | chloral hydrate | Filament; t-MCAO | 30 mg/kg; daily; 7 days | 7 d before ischemia onset | 24 hours | NA | ip |
| 37 | (Orsu et al., 2013) | Rats, NA | thiopental sodium | BCCA | 5/10/20/30 mg/kg; single | 5 min before reperfusion | 4 hours | NA | ip |
| 38 | (Lin et al., 2013) | Rats, male | chloral hydrate | Filament; t-MCAO | 200 mg/kg; daily; 7 days | 7 d before ischemia onset | 24 hours | DMSO | ip |
| 39 | (Lanzillotta et al., 2013) | Mice, male | Isoflurane | Filament; t-MCAO | 680/6800 μg/kg; single | 1 h after ischemia onset | 72 hours | Saline | ip |
| 40 | (Hurtado et al., 2013) | Rats, male | Isoflurane | p-MCAO | 2.5 mg/kg; single | 3 h after ischemia onset | 48 hours | Saline | ip |
| 41 | (Yan et al., 2013) | Rats, NA | NA | Filament; t-MCAO | 50 mg/kg; single | 15 d before ischemia onset | 24 hours | Water | intragastrically |
| 42 | (Shin et al., 2012) | Mice, male | Isoflurane | Filament; t-MCAO | 5 mg/kg; single | 3 h after ischemia onset | 72 hours | ethanol | iv |
| 43 | (Li et al., 2012) | Rats, male | chloral hydrate | Filament; t-MCAO | 30 mg/kg; daily; 4 days | 3 h after reperfusion | 24 hours | DMSO | ip |
| 44 | (Ren et al., 2011) | Rats, male | chloral hydrate | Filament; t-MCAO | 15/30 mg/kg; daily; 7 days | 7 d before ischemia onset | 24 hours | saline | ip |
| 45 | (Shin et al., 2010) | Mice, male and female | Isoflurane | Filament; t-MCAO | 1/2.5/5 mg/kg; single | 3/6 h after ischemia onset | 72 hours | ethanol | iv |
| 46 | (Sakata et al., 2010) | Mice, male | halothane | Filament; t-MCAO | 5/10/20 mg/kg, single; 20 mg/kg, daily for 7 days | 2h/ 7d before ischemia onset | 24 hours | NA | oral gavage |
| 47 | (Li et al., 2010) | Rats, male | sodium pentobarbital | Filament; t-MCAO | 30 mg/kg; daily; 7 days | 7 d before ischemia onset | 24 hours | Corn oil | ip |
| 48 | (Yousuf et al., 2009) | Rats, male | chloral hydrate | Filament; t-MCAO | 10^−7^ g/kg; twice | 15 min before and 2 h after ischemia onset | 24 hours | NA | iv |
| 49 | (Dong et al., 2008) | Mice, male | halothane | Filament; t-MCAO | 50 mg/kg; daily; until day 7 after stroke | 5 min before/ 24 h after/ 72 h after ischemia onset | 7 days | hydroxypropyl h-cyclodextrin | oral gavage |
| 50 | (Tsai et al., 2007) | Rats, male | halothane | Filament; t-MCAO | 1 ug/kg; single | 1 h after ischemia onset | 24 hours | propylene glycol | iv |
| 51 | (Gao et al., 2006) | Mice, male | halothane | Filament; t-MCAO | 50 mg/kg; daily; 7 days | 7 d before ischemia onset | 24 hours | hydroxypropyl h-cyclodextrin | oral gavage |
| 52 | (Inoue, 2003) | Mice, | halothane | p-MCAO | 2/20 mg/kg; single/ daily, 3 days | 1 h/3d before after ischemia onset | 24 hours | NA | oral gavage |
| 53 | (Sinha et al., 2002) | Rats, male | chloral hydrate | Filament; t-MCAO | 20 mg/kg; daily; 21 days | 21 d before ischemia onset | 24 hours | alcohol | ip |
| 54 | (Huang et al., 2001) | Rats, male | halothane | Filament; t-MCAO | 10^−6^/10^−7^/10^−8^/10^−9^ g/kg; single | 15 min before/1 h after ischemia onset | 24 hours | NA | iv |

Abbreviations: BCCA: bi-lateral common carotid artery; p-MCAO: permanent middle cerebral artery occlusion; ip: intraperitoneally; iv: intravenously; NA: not available; t-MCAO: transient middle cerebral artery occlusion.

**Supplementary Table 2. Outcomes of the studies included for the meta-analysis.**

| Sl.no. | Author | Year | Animal numbers  Vehicle | Animal numbers  Resveratrol | Infarct volume | Neurological deficit |
| --- | --- | --- | --- | --- | --- | --- |
| 1 | Yu et al. | 2021 | 5 | 5 | NA | mNSS; Longa score; modified Bederson Score |
| 2 | McDonald et al. | 2021 | 9 | 8 | MRI | Beam Traversal Task; Gait Assessment |
| 3 | Yao et al. | 2020 | 4 | 5 | NA | Rotarod test |
| 4 | Teertam et al. | 2020 | 3 | 3 | TTC | NA |
| 5 | Mota et al. | 2020 | 9-11/11-13 | 9-11/11-13 | Silver-staining | Corner test; Latency-to-move tests |
| 6 | Lu et al. | 2020 | 5 | 4 | NA | mNSS (0-14 point) |
| 7 | Alquisiras-Burgos et al. | 2020 | 6/8 | 6/8 | TTC | limb-use asymmetry test |
| 8 | Pineda-Ramírez et al. | 2020 | 6 | 6 | TTC | NA |
| 9 | Dou et al. | 2019 | 6 | 6 | TTC | 18-point composite neurological score (normal score, 18) |
| 10 | Park et al. | 2019 | 4/8 | 4/8 | TTC | 5-point behavioral scale; Corner test |
| 11 | Grewal et al. | 2019 | 8 | 8 | TTC | Rota Rod test, beam walking test |
| 12 | Yan et al. | 2019 | 10 | 10 | TTC | NA |
| 13 | Liu et al. | 2018 | 5/15 | 5/15 | TTC | mNSS (18-point) |
| 14 | Faggi et al. | 2018 | 6 | 6/7 | TTC | General neurological scale (28-point); Focal neurological scale (28-point) |
| 15 | Hou et al. | 2018 | 5 | 5 | TTC | 5-point behavioral scale |
| 16 | Yu et al. | 2017 | 4 | 4 | TTC | Longa score (0-4); modified Bederson score (0-5); mNSS (0-18) |
| 17 | Koronowski et al. | 2017 | 8 | 6 | TTC | Neurobehavioral battery (0-28 points) |
| 18 | Al Dera | 2017 | 6 | 6 | TTC | NA |
| 19 | He et al. | 2017 | 10 | 10 | TTC | 5 points behavioral scale |
| 20 | Yang et al. | 2016 | 5/10 | 5/10 | TTC | 21-point behavioral scale (normal score, 21) |
| 21 | Wan et al. | 2016 | 4 | 4 | TTC | 5-point behavioral scale |
| 22 | Su et al. | 2016 | 8 | 8 | TTC | 5-point behavioral scale |
| 23 | Lopez et al. | 2016 | 7 | 6 | TTC | NA |
| 24 | Li et al. | 2016 | 6 | 6 | TTC | Morris water maze test |
| 25 | Jeong et al. | 2016 | 14/6 | 13/6 | Cresyl violet-staining | 5-point behavioral scale |
| 26 | Abdel-Aleem et al. | 2016 | 6 | 6 | TTC | NA |
| 27 | Pang et al. | 2015 | 8 | 8 | TTC | 5-point behavioral scale |
| 28 | Pandey et al. | 2015 | 9/8 | 6/8 | TTC | 5-point behavioral scale |
| 29 | Narayanan et al. | 2015 | 8 | 6 | TTC | NA |
| 30 | Koronowski et al. | 2015 | 9-15 | 10-16 | TTC | behavioral scale |
| 31 | Ishrat et al. | 2015 | 6-8 | 6-8 | TTC | 3-point behavioral scale |
| 32 | Hermann et al. | 2015 | 10-12 | 10-14 | Cresyl violet staining | Rota rod; Tight rope; Corner turn tests |
| 33 | Fang et al. | 2015 | 10 | 10 | TTC | 4-point behavioral scale |
| 34 | Li et al. | 2015 | 8 | 8 | TTC | 4-point behavioral scale |
| 35 | Saleh et al. | 2014 | 5 | 5-6 | TTC | NA |
| 36 | Wang et al. | 2014 | 10 | 10 | TTC | 5-point behavioral scale |
| 37 | Orsu et al. | 2013 | 6 | 6 | TTC | NA |
| 38 | Lin et al. | 2013 | 6 | 6 | TTC | 4-point behavioral scale |
| 39 | Lanzillotta et al. | 2013 | 6 | 5-7 | TTC | General neurological scale (28-point) and Focal neurological scale (28-point) |
| 40 | Hurtado et al. | 2013 | 8 | 7 | TTC | NA |
| 41 | Yan et al. | 2013 | 8 | 8 | TTC | 18-point neurological score (normal score, 18) |
| 42 | Shin et al. | 2012 | 8 | 8 | Cresyl violet staining | NA |
| 43 | Li et al. | 2012 | 5 | 4 | TTC | NA |
| 44 | Ren et al. | 2011 | 3/10 | 3/10 | TTC | 5-point behavioral scale |
| 45 | Shin et al. | 2010 | 6 | 4-6 | Cresyl violet staining | NA |
| 46 | Sakata et al. | 2010 | 7 | 5 | TTC | NA |
| 47 | Li et al. | 2010 | 8 | 8 | TTC | 4-point behavioral scale |
| 48 | Yousuf et al. | 2009 | 8 | 8 | TTC | Bederson scores (0-3 point); Spontaneous motor (0-3 point) activity |
| 49 | Dong et al. | 2008 | 8 | 8 | TTC | 5-point behavioral scale |
| 50 | Tsai et al. | 2007 | 12 | 10 | TTC | NA |
| 51 | Gao et al. | 2006 | 10 | 10 | TTC | NA |
| 52 | Inoue et al. | 2003 | 7-10 | 8-10 | TTC | NA |
| 53 | Sinha et al. | 2002 | 7/16 | 7/16 | TTC | Grip test; Rota rod; Spontaneous locomotor activity |
| 54 | Huang et al. | 2001 | 7 | 7 | TTC | NA |

Abbreviations: mNSS: modified neurological severity scores; MRI: Magnetic Resonance Imaging; NA: not available; TTC: 2, 3, 5-triphenyl tetrazolium chloride.

**Supplementary Table 3. Quality scores of the studies included for the meta-analysis.**

| Sl.no. | Author | Year | 1 | 2 | 3 | 4 | 5 | 6 | 7 | 8 | 9 | 10 | Quality score |
| --- | --- | --- | --- | --- | --- | --- | --- | --- | --- | --- | --- | --- | --- |
| 1 | Yu et al. | 2021 | 1 | 0 | 0 | 0 | 0 | 1 | 1 | 0 | 1 | 1 | **5** |
| 2 | McDonald et al. | 2021 | 1 | 1 | 1 | 1 | 0 | 1 | 1 | 0 | 1 | 1 | **8** |
| 3 | Yao et al. | 2020 | 1 | 0 | 1 | 0 | 0 | 0 | 1 | 0 | 1 | 1 | **5** |
| 4 | Teertam et al. | 2020 | 1 | 0 | 1 | 0 | 0 | 0 | 0 | 0 | 1 | 1 | **4** |
| 5 | Mota et al. | 2020 | 1 | 1 | 1 | 0 | 0 | 0 | 1 | 0 | 1 | 1 | **6** |
| 6 | Lu et al. | 2020 | 1 | 1 | 0 | 0 | 0 | 1 | 1 | 0 | 1 | 1 | **6** |
| 7 | Alquisiras-Burgos et al. | 2020 | 1 | 0 | 1 | 0 | 0 | 0 | 1 | 0 | 1 | 0 | **4** |
| 8 | Pineda-Ramírez et al. | 2020 | 1 | 1 | 1 | 0 | 0 | 0 | 1 | 0 | 1 | 0 | **5** |
| 9 | Dou et al. | 2019 | 1 | 0 | 1 | 0 | 0 | 0 | 0 | 0 | 1 | 1 | **4** |
| 10 | Park et al. | 2019 | 1 | 1 | 0 | 0 | 0 | 0 | 1 | 0 | 1 | 1 | **5** |
| 11 | Grewal et al. | 2019 | 1 | 0 | 1 | 0 | 0 | 0 | 1 | 0 | 1 | 1 | **5** |
| 12 | Yan et al. | 2019 | 1 | 0 | 0 | 0 | 0 | 1 | 1 | 0 | 1 | 1 | **5** |
| 13 | Liu et al. | 2018 | 1 | 1 | 1 | 1 | 0 | 1 | 1 | 0 | 1 | 0 | **7** |
| 14 | Faggi et al. | 2018 | 1 | 1 | 1 | 1 | 0 | 1 | 1 | 0 | 1 | 1 | **8** |
| 15 | Hou et al. | 2018 | 1 | 1 | 1 | 1 | 0 | 1 | 1 | 0 | 0 | 1 | **7** |
| 16 | Yu et al. | 2017 | 1 | 1 | 0 | 0 | 0 | 1 | 1 | 0 | 1 | 1 | **6** |
| 17 | Koronowski et al. | 2017 | 1 | 1 | 1 | 1 | 0 | 1 | 1 | 0 | 1 | 0 | **7** |
| 18 | Al Dera | 2017 | 1 | 1 | 0 | 0 | 0 | 0 | 1 | 0 | 1 | 1 | **5** |
| 19 | He et al. | 2017 | 1 | 1 | 0 | 0 | 0 | 1 | 1 | 0 | 1 | 0 | **5** |
| 20 | Yang et al. | 2016 | 1 | 1 | 1 | 1 | 0 | 1 | 1 | 0 | 1 | 0 | **7** |
| 21 | Wan et al. | 2016 | 1 | 1 | 1 | 1 | 0 | 1 | 1 | 0 | 1 | 0 | **7** |
| 22 | Su et al. | 2016 | 1 | 0 | 1 | 1 | 0 | 1 | 1 | 0 | 1 | 1 | **7** |
| 23 | Lopez et al. | 2016 | 1 | 0 | 1 | 1 | 0 | 1 | 1 | 0 | 1 | 1 | **7** |
| 24 | Li et al. | 2016 | 1 | 1 | 0 | 0 | 0 | 1 | 1 | 0 | 1 | 0 | **5** |
| 25 | Jeong et al. | 2016 | 1 | 1 | 1 | 1 | 1 | 1 | 1 | 0 | 1 | 1 | **9** |
| 26 | Abdel-Aleem et al. | 2016 | 1 | 1 | 1 | 0 | 0 | 0 | 1 | 0 | 1 | 1 | **6** |
| 27 | Pang et al. | 2015 | 1 | 1 | 1 | 0 | 0 | 0 | 1 | 0 | 1 | 1 | **6** |
| 28 | Pandey et al. | 2015 | 1 | 0 | 1 | 0 | 0 | 0 | 0 | 0 | 1 | 1 | **4** |
| 29 | Narayanan et al. | 2015 | 1 | 1 | 1 | 1 | 0 | 1 | 1 | 0 | 1 | 0 | **7** |
| 30 | Koronowski et al. | 2015 | 1 | 1 | 1 | 1 | 0 | 1 | 1 | 0 | 1 | 0 | **7** |
| 31 | Ishrat et al. | 2015 | 1 | 1 | 0 | 0 | 0 | 1 | 1 | 0 | 1 | 1 | **6** |
| 32 | Hermann et al. | 2015 | 1 | 0 | 1 | 1 | 0 | 1 | 1 | 0 | 1 | 1 | **7** |
| 33 | Fang et al. | 2015 | 1 | 1 | 1 | 1 | 0 | 1 | 1 | 0 | 1 | 1 | **8** |
| 34 | Li et al. | 2015 | 1 | 0 | 1 | 0 | 0 | 0 | 1 | 0 | 1 | 0 | **4** |
| 35 | Saleh et al. | 2014 | 1 | 1 | 0 | 0 | 0 | 0 | 1 | 0 | 1 | 1 | **5** |
| 36 | Wang et al. | 2014 | 1 | 1 | 1 | 1 | 0 | 1 | 1 | 0 | 1 | 0 | **7** |
| 37 | Orsu et al. | 2013 | 1 | 1 | 1 | 0 | 0 | 0 | 1 | 0 | 1 | 1 | **6** |
| 38 | Lin et al. | 2013 | 1 | 1 | 1 | 0 | 0 | 0 | 1 | 0 | 1 | 1 | **6** |
| 39 | Lanzillotta et al. | 2013 | 1 | 0 | 1 | 1 | 0 | 1 | 1 | 0 | 1 | 1 | **8** |
| 40 | Hurtado et al. | 2013 | 1 | 1 | 1 | 1 | 0 | 1 | 1 | 0 | 1 | 1 | **8** |
| 41 | Yan et al. | 2013 | 1 | 0 | 0 | 0 | 0 | 0 | 1 | 0 | 1 | 1 | **4** |
| 42 | Shin et al. | 2012 | 1 | 1 | 1 | 0 | 0 | 0 | 1 | 0 | 1 | 1 | **6** |
| 43 | Li et al. | 2012 | 1 | 1 | 1 | 1 | 0 | 1 | 1 | 0 | 1 | 0 | **7** |
| 44 | Ren et al. | 2011 | 1 | 1 | 1 | 1 | 0 | 1 | 1 | 0 | 1 | 0 | **7** |
| 45 | Shin et al. | 2010 | 1 | 1 | 1 | 1 | 0 | 1 | 1 | 0 | 1 | 0 | **7** |
| 46 | Sakata et al. | 2010 | 1 | 1 | 0 | 0 | 0 | 0 | 1 | 0 | 1 | 0 | **4** |
| 47 | Li et al. | 2010 | 1 | 1 | 1 | 0 | 0 | 0 | 1 | 0 | 1 | 0 | **5** |
| 48 | Yousuf et al. | 2009 | 1 | 1 | 0 | 0 | 0 | 0 | 1 | 0 | 1 | 0 | **4** |
| 49 | Dong et al. | 2008 | 1 | 1 | 1 | 1 | 0 | 1 | 1 | 0 | 0 | 1 | **7** |
| 50 | Tsai et al. | 2007 | 1 | 1 | 1 | 1 | 0 | 1 | 1 | 0 | 1 | 1 | **8** |
| 51 | Gao et al. | 2006 | 1 | 1 | 0 | 0 | 0 | 0 | 1 | 0 | 0 | 0 | **3** |
| 52 | Inoue et al. | 2003 | 1 | 1 | 0 | 0 | 0 | 1 | 1 | 0 | 0 | 0 | **4** |
| 53 | Sinha et al. | 2002 | 1 | 1 | 0 | 0 | 0 | 0 | 1 | 0 | 1 | 0 | **4** |
| 54 | Huang et al. | 2001 | 1 | 1 | 1 | 1 | 0 | 1 | 1 | 0 | 1 | 0 | **7** |

(1) publication in a peer-reviewed journal, (2) statements describing temperature control, (3) randomized treatment allocation, (4) allocation csinglealment, (5) use of animal models (aged, diabetic, or hypertensive), (6) blind assessment of outcome, (7) avoidance of anesthetics with significant intrinsic neuroprotective activity such as ketamine, (8) reporting of a sample size calculation, (9) statement of compliance with regulatory requirements, and (10) declarations of potential conflicts of interest.

**References**

Abdel-Aleem, G.A., Khaleel, E.F., Mostafa, D.G., and Elberier, L.K. (2016). Neuroprotective effect of resveratrol against brain ischemia reperfusion injury in rats entails reduction of DJ-1 protein expression and activation of PI3K/Akt/GSK3b survival pathway. *Arch Physiol Biochem* 122(4)**,** 200-213. doi: 10.1080/13813455.2016.1182190.

Al Dera, H. (2017). Neuroprotective effect of resveratrol against late cerebral ischemia reperfusion induced oxidative stress damage involves upregulation of osteopontin and inhibition of interleukin-1beta. *J Physiol Pharmacol* 68(1)**,** 47-56.

Alquisiras-Burgos, I., Ortiz-Plata, A., Franco-Pérez, J., Millán, A., and Aguilera, P. (2020). Resveratrol reduces cerebral edema through inhibition of de novo SUR1 expression induced after focal ischemia. *Exp Neurol* 330**,** 113353. doi: 10.1016/j.expneurol.2020.113353.

Dong, W., Li, N., Gao, D., Zhen, H., Zhang, X., and Li, F. (2008). Resveratrol attenuates ischemic brain damage in the delayed phase after stroke and induces messenger RNA and protein express for angiogenic factors. *J Vasc Surg* 48(3)**,** 709-714. doi: 10.1016/j.jvs.2008.04.007.

Dou, Z., Rong, X., Zhao, E., Zhang, L., and Lv, Y. (2019). Neuroprotection of Resveratrol Against Focal Cerebral Ischemia/Reperfusion Injury in Mice Through a Mechanism Targeting Gut-Brain Axis. *Cell Mol Neurobiol* 39(6)**,** 883-898. doi: 10.1007/s10571-019-00687-3.

Faggi, L., Pignataro, G., Parrella, E., Porrini, V., Vinciguerra, A., Cepparulo, P., et al. (2018). Synergistic Association of Valproate and Resveratrol Reduces Brain Injury in Ischemic Stroke. *Int J Mol Sci* 19(1). doi: 10.3390/ijms19010172.

Fang, L., Gao, H., Zhang, W., Zhang, W., and Wang, Y. (2015). Resveratrol alleviates nerve injury after cerebral ischemia and reperfusion in mice by inhibiting inflammation and apoptosis. *Int J Clin Exp Med* 8(3)**,** 3219-3226.

Gao, D., Zhang, X., Jiang, X., Peng, Y., Huang, W., Cheng, G., et al. (2006). Resveratrol reduces the elevated level of MMP-9 induced by cerebral ischemia-reperfusion in mice. *Life Sci* 78(22)**,** 2564-2570. doi: 10.1016/j.lfs.2005.10.030.

Grewal, A.K., Singh, N., and Singh, T.G. (2019). Effects of resveratrol postconditioning on cerebral ischemia in mice: role of the sirtuin-1 pathway. *Can J Physiol Pharmacol* 97(11)**,** 1094-1101. doi: 10.1139/cjpp-2019-0188.

He, Q., Li, Z., Wang, Y., Hou, Y., Li, L., and Zhao, J. (2017). Resveratrol alleviates cerebral ischemia/reperfusion injury in rats by inhibiting NLRP3 inflammasome activation through Sirt1-dependent autophagy induction. *Int Immunopharmacol* 50**,** 208-215. doi: 10.1016/j.intimp.2017.06.029.

Hermann, D.M., Zechariah, A., Kaltwasser, B., Bosche, B., Caglayan, A.B., Kilic, E., et al. (2015). Sustained neurological recovery induced by resveratrol is associated with angioneurogenesis rather than neuroprotection after focal cerebral ischemia. *Neurobiol Dis* 83**,** 16-25. doi: 10.1016/j.nbd.2015.08.018.

Hou, Y., Wang, K., Wan, W., Cheng, Y., Pu, X., and Ye, X. (2018). Resveratrol provides neuroprotection by regulating the JAK2/STAT3/PI3K/AKT/mTOR pathway after stroke in rats. *Genes Dis* 5(3)**,** 245-255. doi: 10.1016/j.gendis.2018.06.001.

Huang, S.S., Tsai, M.C., Chih, C.L., Hung, L.M., and Tsai, S.K. (2001). Resveratrol reduction of infarct size in Long-Evans rats subjected to focal cerebral ischemia. *Life Sci* 69(9)**,** 1057-1065. doi: 10.1016/s0024-3205(01)01195-x.

Hurtado, O., Hernández-Jiménez, M., Zarruk, J.G., Cuartero, M.I., Ballesteros, I., Camarero, G., et al. (2013). Citicoline (CDP-choline) increases Sirtuin1 expression concomitant to neuroprotection in experimental stroke. *J Neurochem* 126(6)**,** 819-826. doi: 10.1111/jnc.12269.

Inoue, H. (2003). Brain protection by resveratrol and fenofibrate against stroke requires peroxisome proliferator-activated receptor α in mice. *Neuroscience Letters*. doi: 10.1016/s0304-3940(03)01080-2.

Ishrat, T., Mohamed, I.N., Pillai, B., Soliman, S., Fouda, A.Y., Ergul, A., et al. (2015). Thioredoxin-interacting protein: a novel target for neuroprotection in experimental thromboembolic stroke in mice. *Mol Neurobiol* 51(2)**,** 766-778. doi: 10.1007/s12035-014-8766-x.

Jeong, S.I., Shin, J.A., Cho, S., Kim, H.W., Lee, J.Y., Kang, J.L., et al. (2016). Resveratrol attenuates peripheral and brain inflammation and reduces ischemic brain injury in aged female mice. *Neurobiol Aging* 44**,** 74-84. doi: 10.1016/j.neurobiolaging.2016.04.007.

Koronowski, K.B., Dave, K.R., Saul, I., Camarena, V., Thompson, J.W., Neumann, J.T., et al. (2015). Resveratrol Preconditioning Induces a Novel Extended Window of Ischemic Tolerance in the Mouse Brain. *Stroke* 46(8)**,** 2293-2298. doi: 10.1161/strokeaha.115.009876.

Koronowski, K.B., Khoury, N., Saul, I., Loris, Z.B., Cohan, C.H., Stradecki-Cohan, H.M., et al. (2017). Neuronal SIRT1 (Silent Information Regulator 2 Homologue 1) Regulates Glycolysis and Mediates Resveratrol-Induced Ischemic Tolerance. *Stroke* 48(11)**,** 3117-3125. doi: 10.1161/strokeaha.117.018562.

Lanzillotta, A., Pignataro, G., Branca, C., Cuomo, O., Sarnico, I., Benarese, M., et al. (2013). Targeted acetylation of NF-kappaB/RelA and histones by epigenetic drugs reduces post-ischemic brain injury in mice with an extended therapeutic window. *Neurobiol Dis* 49**,** 177-189. doi: 10.1016/j.nbd.2012.08.018.

Li, C., Yan, Z., Yang, J., Chen, H., Li, H., Jiang, Y., et al. (2010). Neuroprotective effects of resveratrol on ischemic injury mediated by modulating the release of neurotransmitter and neuromodulator in rats. *Neurochem Int* 56(3)**,** 495-500. doi: 10.1016/j.neuint.2009.12.009.

Li, W., Tan, C., Liu, Y., Liu, X., Wang, X., Gui, Y., et al. (2015). Resveratrol ameliorates oxidative stress and inhibits aquaporin 4 expression following rat cerebral ischemia-reperfusion injury. *Mol Med Rep* 12(5)**,** 7756-7762. doi: 10.3892/mmr.2015.4366.

Li, Z., Fang, F., Wang, Y., and Wang, L. (2016). Resveratrol protects CA1 neurons against focal cerebral ischemic reperfusion-induced damage via the ERK-CREB signaling pathway in rats. *Pharmacol Biochem Behav* 146-147**,** 21-27. doi: 10.1016/j.pbb.2016.04.007.

Li, Z., Pang, L., Fang, F., Zhang, G., Zhang, J., Xie, M., et al. (2012). Resveratrol attenuates brain damage in a rat model of focal cerebral ischemia via up-regulation of hippocampal Bcl-2. *Brain Res* 1450**,** 116-124. doi: 10.1016/j.brainres.2012.02.019.

Lin, Y., Chen, F., Zhang, J., Wang, T., Wei, X., Wu, J., et al. (2013). Neuroprotective effect of resveratrol on ischemia/reperfusion injury in rats through TRPC6/CREB pathways. *J Mol Neurosci* 50(3)**,** 504-513. doi: 10.1007/s12031-013-9977-8.

Liu, Y., Yang, H., Jia, G., Li, L., Chen, H., Bi, J., et al. (2018). The Synergistic Neuroprotective Effects of Combined Rosuvastatin and Resveratrol Pretreatment against Cerebral Ischemia/Reperfusion Injury. *J Stroke Cerebrovasc Dis* 27(6)**,** 1697-1704. doi: 10.1016/j.jstrokecerebrovasdis.2018.01.033.

Lopez, M.S., Dempsey, R.J., and Vemuganti, R. (2016). Resveratrol preconditioning induces cerebral ischemic tolerance but has minimal effect on cerebral microRNA profiles. *J Cereb Blood Flow Metab* 36(9)**,** 1644-1650. doi: 10.1177/0271678x16656202.

Lu, X., Dong, J., Zheng, D., Li, X., Ding, D., and Xu, H. (2020). Reperfusion combined with intraarterial administration of resveratrol-loaded nanoparticles improved cerebral ischemia-reperfusion injury in rats. *Nanomedicine* 28**,** 102208. doi: 10.1016/j.nano.2020.102208.

McDonald, M.W., Jeffers, M.S., Issa, L., Carter, A., Ripley, A., Kuhl, L.M., et al. (2021). An Exercise Mimetic Approach to Reduce Poststroke Deconditioning and Enhance Stroke Recovery. *Neurorehabil Neural Repair* 35(6)**,** 471-485. doi: 10.1177/15459683211005019.

Mota, M., Porrini, V., Parrella, E., Benarese, M., Bellucci, A., Rhein, S., et al. (2020). Neuroprotective epi-drugs quench the inflammatory response and microglial/macrophage activation in a mouse model of permanent brain ischemia. *J Neuroinflammation* 17(1)**,** 361. doi: 10.1186/s12974-020-02028-4.

Narayanan, S.V., Dave, K.R., Saul, I., and Perez-Pinzon, M.A. (2015). Resveratrol Preconditioning Protects Against Cerebral Ischemic Injury via Nuclear Erythroid 2-Related Factor 2. *Stroke* 46(6)**,** 1626-1632. doi: 10.1161/strokeaha.115.008921.

Orsu, P., Murthy, B.V., and Akula, A. (2013). Cerebroprotective potential of resveratrol through anti-oxidant and anti-inflammatory mechanisms in rats. *J Neural Transm (Vienna)* 120(8)**,** 1217-1223. doi: 10.1007/s00702-013-0982-4.

Pandey, A.K., Bhattacharya, P., Shukla, S.C., Paul, S., and Patnaik, R. (2015). Resveratrol inhibits matrix metalloproteinases to attenuate neuronal damage in cerebral ischemia: a molecular docking study exploring possible neuroprotection. *Neural Regen Res* 10(4)**,** 568-575. doi: 10.4103/1673-5374.155429.

Pang, C., Cao, L., Wu, F., Wang, L., Wang, G., Yu, Y., et al. (2015). The effect of trans-resveratrol on post-stroke depression via regulation of hypothalamus-pituitary-adrenal axis. *Neuropharmacology* 97**,** 447-456. doi: 10.1016/j.neuropharm.2015.04.017.

Park, D.-J., Kang, J.-B., Shah, F.-A., and Koh, P.-O. (2019). Resveratrol modulates the Akt/GSK-3beta signaling pathway in a middle cerebral artery occlusion animal model. *Laboratory animal research* 35**,** 18-18. doi: 10.1186/s42826-019-0019-8.

Pineda-Ramírez, N., Alquisiras-Burgos, I., Ortiz-Plata, A., Ruiz-Tachiquín, M.E., Espinoza-Rojo, M., and Aguilera, P. (2020). Resveratrol Activates Neuronal Autophagy Through AMPK in the Ischemic Brain. *Mol Neurobiol* 57(2)**,** 1055-1069. doi: 10.1007/s12035-019-01803-6.

Ren, J., Fan, C., Chen, N., Huang, J., and Yang, Q. (2011). Resveratrol pretreatment attenuates cerebral ischemic injury by upregulating expression of transcription factor Nrf2 and HO-1 in rats. *Neurochem Res* 36(12)**,** 2352-2362. doi: 10.1007/s11064-011-0561-8.

Sakata, Y., Zhuang, H., Kwansa, H., Koehler, R.C., and Dore, S. (2010). Resveratrol protects against experimental stroke: putative neuroprotective role of heme oxygenase 1. *Exp Neurol* 224(1)**,** 325-329. doi: 10.1016/j.expneurol.2010.03.032.

Saleh, M.C., Connell, B.J., Rajagopal, D., Khan, B.V., Abd-El-Aziz, A.S., Kucukkaya, I., et al. (2014). Co-administration of resveratrol and lipoic acid, or their synthetic combination, enhances neuroprotection in a rat model of ischemia/reperfusion. *PLoS One* 9(1)**,** e87865. doi: 10.1371/journal.pone.0087865.

Shin, J.A., Lee, H., Lim, Y.K., Koh, Y., Choi, J.H., and Park, E.M. (2010). Therapeutic effects of resveratrol during acute periods following experimental ischemic stroke. *J Neuroimmunol* 227(1-2)**,** 93-100. doi: 10.1016/j.jneuroim.2010.06.017.

Shin, J.A., Lee, K.E., Kim, H.S., and Park, E.M. (2012). Acute resveratrol treatment modulates multiple signaling pathways in the ischemic brain. *Neurochem Res* 37(12)**,** 2686-2696. doi: 10.1007/s11064-012-0858-2.

Sinha, K., Chaudhary, G., and Gupta, Y.K. (2002). Protective effect of resveratrol against oxidative stress in middle cerebral artery occlusion model of stroke in rats. *Life Sci* 71(6)**,** 655-665. doi: 10.1016/s0024-3205(02)01691-0.

Su, Q., Pu, H., and Hu, C. (2016). Neuroprotection by combination of resveratrol and enriched environment against ischemic brain injury in rats. *Neurol Res* 38(1)**,** 60-68. doi: 10.1080/01616412.2015.1133027.

Teertam, S.K., Jha, S., and Prakash Babu, P. (2020). Up-regulation of Sirt1/miR-149-5p signaling may play a role in resveratrol induced protection against ischemia via p53 in rat brain. *J Clin Neurosci* 72**,** 402-411. doi: 10.1016/j.jocn.2019.11.043.

Tsai, S.K., Hung, L.M., Fu, Y.T., Cheng, H., Nien, M.W., Liu, H.Y., et al. (2007). Resveratrol neuroprotective effects during focal cerebral ischemia injury via nitric oxide mechanism in rats. *J Vasc Surg* 46(2)**,** 346-353. doi: 10.1016/j.jvs.2007.04.044.

Wan, D., Zhou, Y., Wang, K., Hou, Y., Hou, R., and Ye, X. (2016). Resveratrol provides neuroprotection by inhibiting phosphodiesterases and regulating the cAMP/AMPK/SIRT1 pathway after stroke in rats. *Brain Res Bull* 121**,** 255-262. doi: 10.1016/j.brainresbull.2016.02.011.

Wang, R., Liu, Y.Y., Liu, X.Y., Jia, S.W., Zhao, J., Cui, D., et al. (2014). Resveratrol protects neurons and the myocardium by reducing oxidative stress and ameliorating mitochondria damage in a cerebral ischemia rat model. *Cell Physiol Biochem* 34(3)**,** 854-864. doi: 10.1159/000366304.

Yan, W., Fang, Z., Yang, Q., Dong, H., Lu, Y., Lei, C., et al. (2013). SirT1 mediates hyperbaric oxygen preconditioning-induced ischemic tolerance in rat brain. *J Cereb Blood Flow Metab* 33(3)**,** 396-406. doi: 10.1038/jcbfm.2012.179.

Yan, Y., Tong, F., and Chen, J. (2019). Endogenous BMP-4/ROS/COX-2 Mediated IPC and Resveratrol Alleviated Brain Damage. *Curr Pharm Des* 25(9)**,** 1030-1039. doi: 10.2174/1381612825666190506120611.

Yang, H., Zhang, A., Zhang, Y., Ma, S., and Wang, C. (2016). Resveratrol Pretreatment Protected against Cerebral Ischemia/Reperfusion Injury in Rats via Expansion of T Regulatory Cells. *J Stroke Cerebrovasc Dis* 25(8)**,** 1914-1921. doi: 10.1016/j.jstrokecerebrovasdis.2016.04.014.

Yao, Y., Zhou, R., Bai, R., Wang, J., Tu, M., Shi, J., et al. (2020). Resveratrol promotes the survival and neuronal differentiation of hypoxia-conditioned neuronal progenitor cells in rats with cerebral ischemia. *Front Med*. doi: 10.1007/s11684-021-0832-y.

Yousuf, S., Atif, F., Ahmad, M., Hoda, N., Ishrat, T., Khan, B., et al. (2009). Resveratrol exerts its neuroprotective effect by modulating mitochondrial dysfunctions and associated cell death during cerebral ischemia. *Brain Res* 1250**,** 242-253. doi: 10.1016/j.brainres.2008.10.068.

Yu, P., Wang, L., Tang, F., Guo, S., Liao, H., Fan, C., et al. (2021). Resveratrol-mediated neurorestoration after cerebral ischemic injury - Sonic Hedgehog signaling pathway. *Life Sci* 280**,** 119715. doi: 10.1016/j.lfs.2021.119715.

Yu, P., Wang, L., Tang, F., Zeng, L., Zhou, L., Song, X., et al. (2017). Resveratrol Pretreatment Decreases Ischemic Injury and Improves Neurological Function Via Sonic Hedgehog Signaling After Stroke in Rats. *Mol Neurobiol* 54(1)**,** 212-226. doi: 10.1007/s12035-015-9639-7.
